# Type II Pleuropulmonary Blastoma in a Two-Year-Old Girl: A Case Report and Literature Review

**DOI:** 10.7759/cureus.72253

**Published:** 2024-10-24

**Authors:** Evenildo Martinez Ortega, Dollis De Jesús Rodríguez Ruano, Mohammed Ajebli, Amani N Al-Ansari

**Affiliations:** 1 Surgery, Hamad Medical Corporation, Doha, QAT; 2 Pediatric Surgery, Juan Manuel Marquez Pediatric Hospital, Havana, CUB; 3 Biological Sciences, Faculty of Sciences and Technologies, Moulay Ismail University, Errachidia, MAR; 4 Pediatric Surgery, Hamad Medical Corporation, Doha, QAT

**Keywords:** case report, diagnosis, pediatric patients, pleuropulmonary blastoma (ppb), surgical treatment

## Abstract

Pleuropulmonary blastoma (PPB) is an extremely rare and highly malignant intrathoracic tumor in children, representing a unique form of aggressive primary lung carcinoma with a strong tendency for local recurrence. In this case report, we present a two-year-old girl who has had recurrent respiratory infections since birth. A chest X-ray revealed an abnormality, prompting a referral to a surgical team, where the lesion was identified as type II PPB based on histological analysis. After completing polychemotherapy, the patient was monitored for five years, during which clinical exams and imaging showed no evidence of disease. Diagnosing PPB requires a high index of suspicion due to its aggressive yet subtle presentation, as imaging findings and clinical symptoms may be nonspecific. This report emphasizes the diagnostic and management challenges of type II PPB, underscoring the importance of a multidisciplinary approach in low-resource settings. We explore potential prognostic factors, discuss treatment options, and propose individualized therapeutic strategies.

## Introduction

Pleuropulmonary blastoma (PPB) was first identified by Barnard [1] as a rare and highly aggressive primary lung carcinoma with a strong propensity for local recurrence. It is classified under the World Health Organization-International Classification of Diseases for Oncology with the code 8973/3. PPB type II predominantly affects the lungs and pleura and accounts for approximately 90% of all pediatric lung cancers. First described in the literature in 1988, PPB type II has been recognized as a distinct entity with unique clinical, pathological, and radiological features. Early diagnosis and prompt management are critical for improving patient outcomes due to its aggressive nature. PPB may originate in the lung, pleura, or both and accounts for 5% of all childhood cancers, disproportionately affecting children under the age of 5. Notably, 90% of cases occur within the first two years of life. Unlike the traditional adult form, PPB exhibits blastomatous and sarcomatous characteristics without an epithelial component, resembling dysembryonic tumors such as Wilms tumors and neuroblastomas. Histological classification follows the Dehner system, with type I being cystic, type II intermediate, and type III solid [2-4].

PPB frequently metastasizes to the brain, and the histological appearance of metastatic lesions tends to be more uniform than the varied morphology of the primary tumor. Therefore, brain imaging is essential in patients with PPB [5,6]. Individuals with PPB also have a higher incidence of rare extrapulmonary benign or malignant neoplastic conditions, such as ovarian and renal cystic nephroma, Sertoli-Leydig tumors, nodular hyperplasia, and thyroid carcinoma [7]. Ribonuclease III (DICER1) plays a key role in post-transcriptional gene expression regulation by generating microRNAs and small interfering RNAs that modulate gene expression. Germline DICER1 mutations are found in approximately 69-80% of patients, supporting the need for DICER1 mutation screening in symptomatic cases [8,9].

This case report presents a two-year-old girl diagnosed with a right thoracic mass, which was surgically removed. We discuss this case in the context of the existing literature. Notably, this patient is the only survivor among three similar cases treated in Cuba. The primary aim of this report is to underscore the importance of timely diagnosis and treatment of thoracic lesions in young patients. This study is particularly unique in presenting a case of PPB treated in a resource-limited setting.

## Case presentation

**Figure 1:**
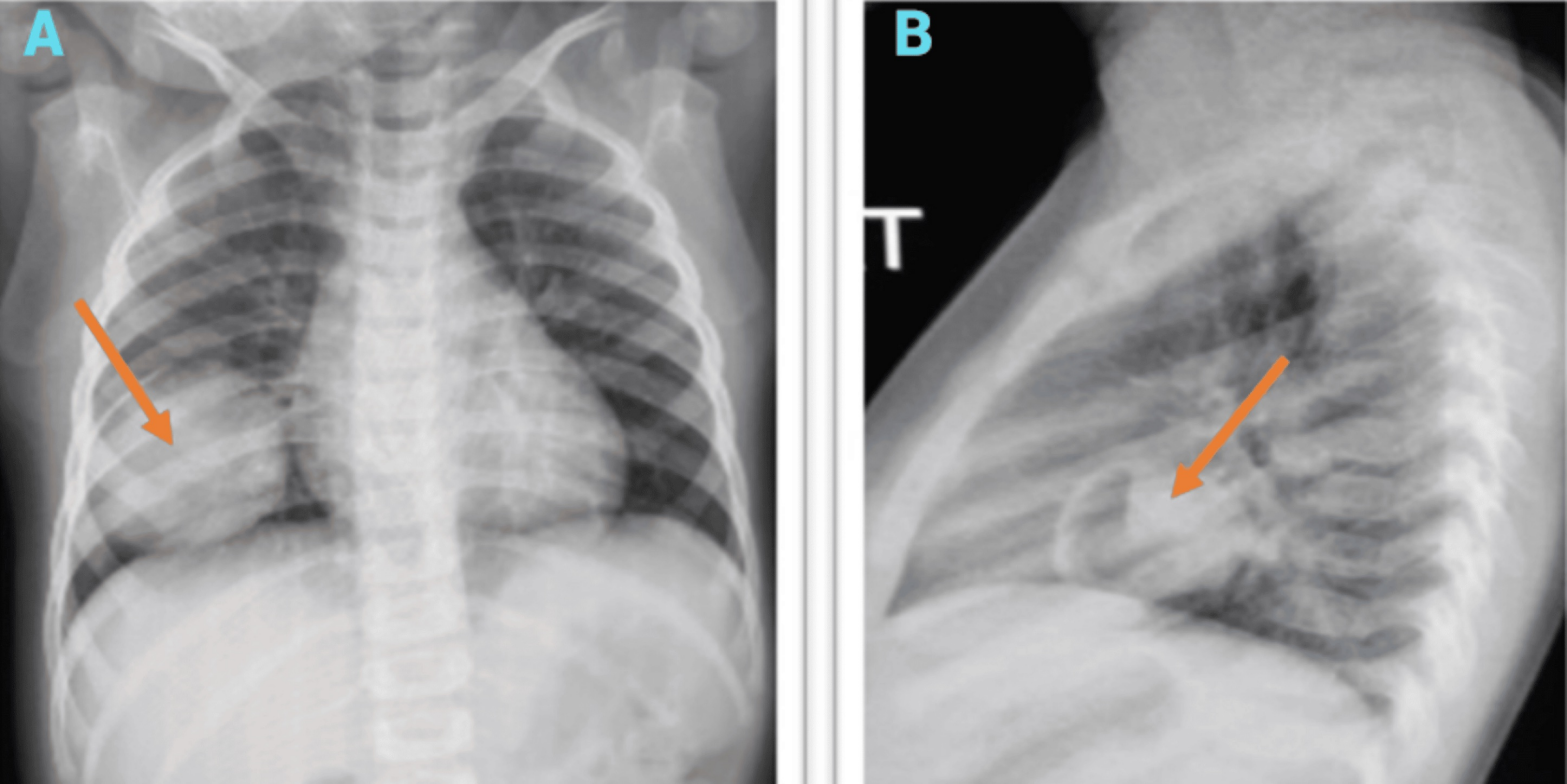
(A) AP chest X-ray showing a homogeneous pulmonary mass at the base of the right hemithorax with regular contours, measuring 6 cm in diameter. (B) Lateral view demonstrating a cystic component located in the RML. RML, right middle lobe

A two-year-old female infant was admitted primarily due to fever, cough, and loss of appetite. She was provisionally diagnosed with an upper respiratory tract infection and had a history of recurrent respiratory illnesses since birth. During the evaluation, a mass was unexpectedly discovered on the chest X-ray, prompting a consultation with the pediatric surgery team. Dyspnea was not evident, and the patient’s chest wall mobility was intact. Auscultation revealed a relative decrease in breath sounds on the right side of the chest. The chest X-ray showed a circular, homogeneous opacity in the middle lobe of the right lung (Figure 1), with no mediastinal displacement.

**Figure 2:**
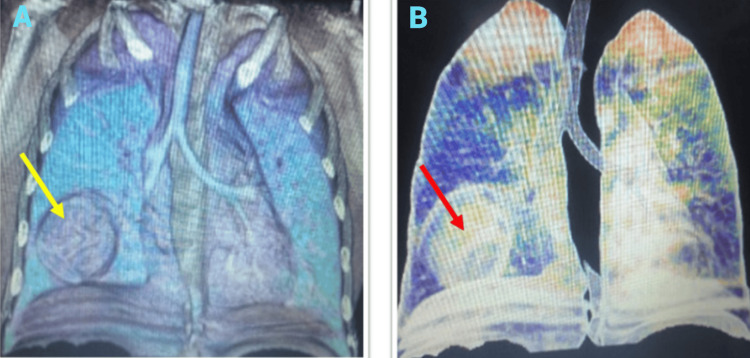
(A) CT scan of the thorax with IV contrast showing a mixed solid and cystic lesion localized in the RML, with no evidence of metastases in the mediastinum, bones, lymph nodes, or pleura. (B) 3D reconstruction illustrating the same findings. RML, right middle lobe

Due to the absence of a local CT scanner, the patient was referred to another facility for a chest CT scan with IV contrast (Figure 2). The scan revealed a heterogeneous, well-delineated mass measuring approximately 6 cm, located in the right lung middle lobe, without compressing adjacent lung tissue. No metastases were detected in the chest or mediastinum. Additional testing, including an echocardiogram, showed no abnormalities. Blood tests (complete blood count, coagulation profile, arterial blood gas, sputum culture, and kidney and liver function tests) were within normal limits. Genetic testing indicated no hereditary issues in the family, including a complete absence of DICER1 mutation history. This tumor represented the primary condition in the spectrum of DICER1-related tumors, such as ovarian sex cord-stromal tumors and thyroid gland nodular hyperplasia. All associated symptoms were inquired about; however, the decision not to conduct the DICER1 gene test was influenced by its financial cost.

The patient underwent excision of the mass and has been monitored oncologically for five years. Based on radiological findings, a thoracotomy and total excision of the tumor were performed. Currently, she is eight years old and asymptomatic.

Treatment

**Figure 3:**
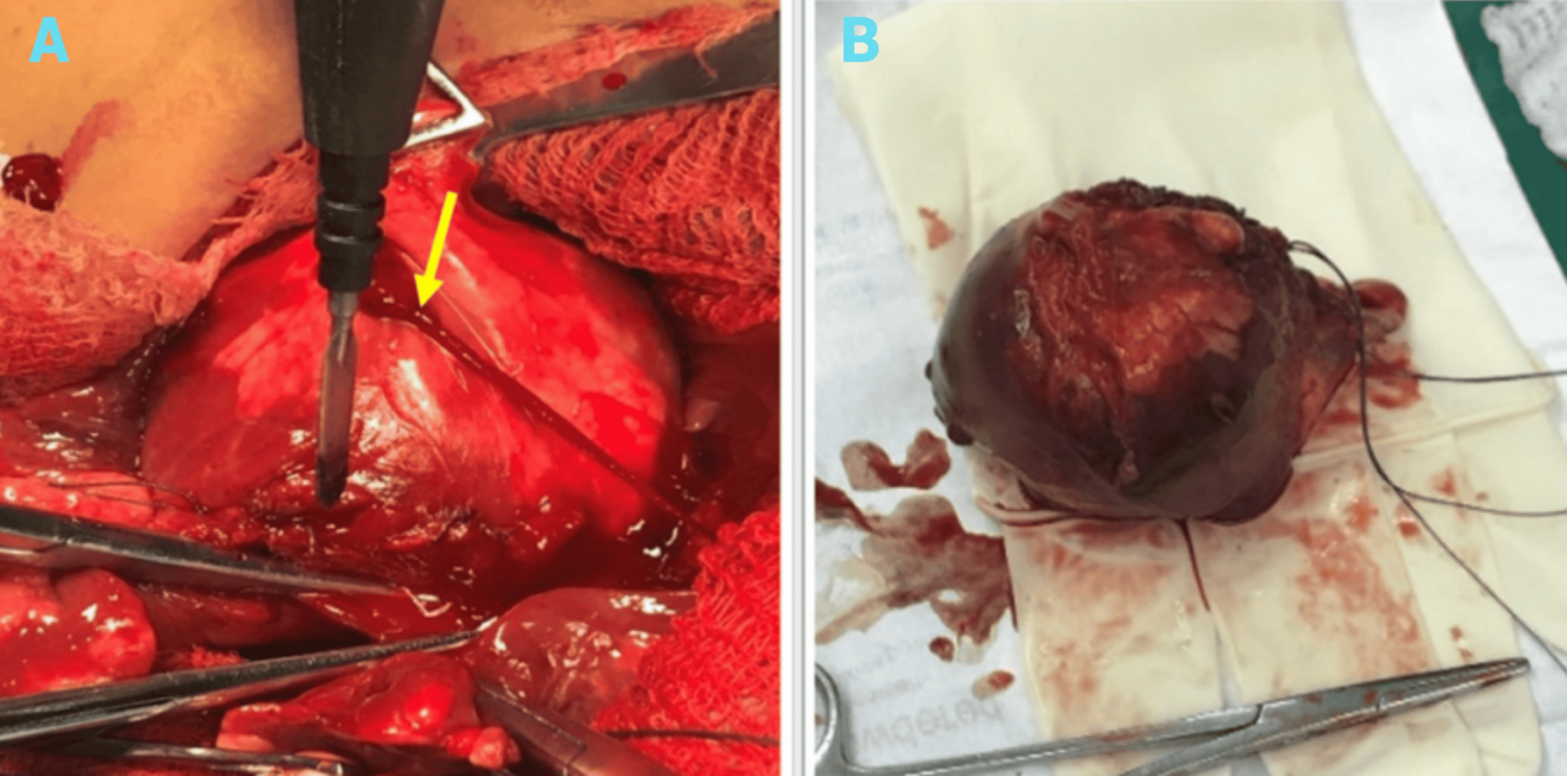
(A) Right anterior lateral thoracotomy revealing the mass in the RML of the lung. (B) Complete excision of the mass, measuring 6 × 4.5 × 6 cm. The yellow arrow indicates the mass in the RML. RML, right middle lobe

An anterolateral thoracotomy was performed in the fourth intercostal space to access the right thoracic cavity, as illustrated in Figure 3. Upon opening the parietal pleura, a mass was identified in the middle lobe of the right lung. This mass was not attached to the chest wall and was anatomically distinct from the surrounding lung parenchyma. Extensive adhesiolysis was followed by dissection and complete excision of the mass, ensuring a free margin of 3 cm all around. Hemostasis was achieved, and an intercostal nerve block was administered using bupivacaine 0.5% from the third to fifth intercostal spaces. A size 22-Fr chest tube was then inserted.

**Figure 4:**
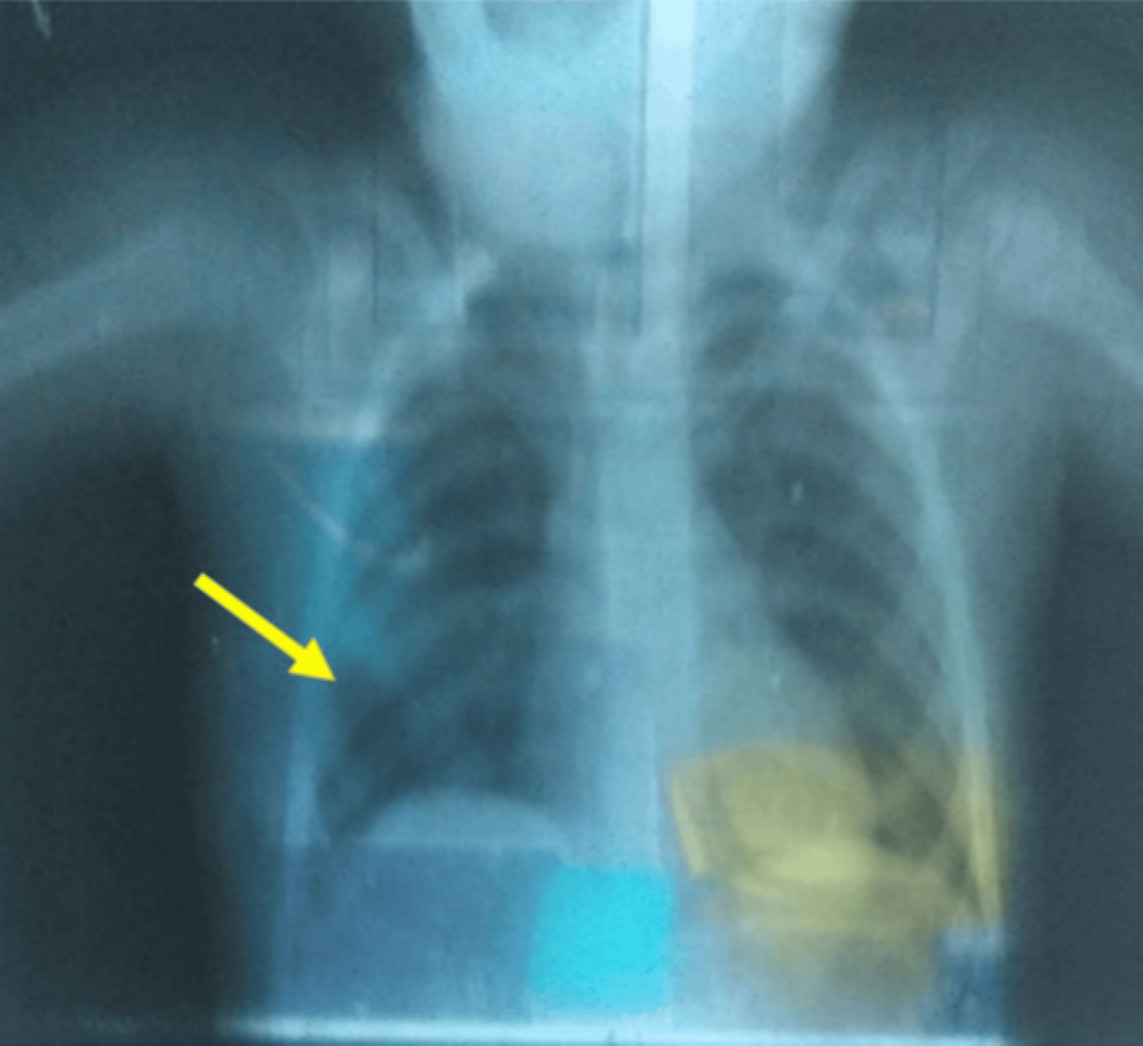
Immediate postoperative chest X-ray showing full expansion of the right lung. The yellow area in the left lower lung indicates postoperative lung expansion following surgery.

The surgical course was uneventful, and the patient was discharged from the hospital seven days post-surgery in stable condition. Within a month, she returned to the clinic without any signs of surgical complications. Follow-up chest X-rays demonstrated complete expansion of the right lung (Figure 4). The patient subsequently received adjuvant polychemotherapy and continued follow-ups with pediatric oncology. The surgical team conducted quarterly checkups for the first two years, with blood tests and X-rays every six months, along with an annual CT scan to monitor her condition. The patient has been under the care of an oncologist for the past five years. 

Histopathological findings

**Figure 5:**
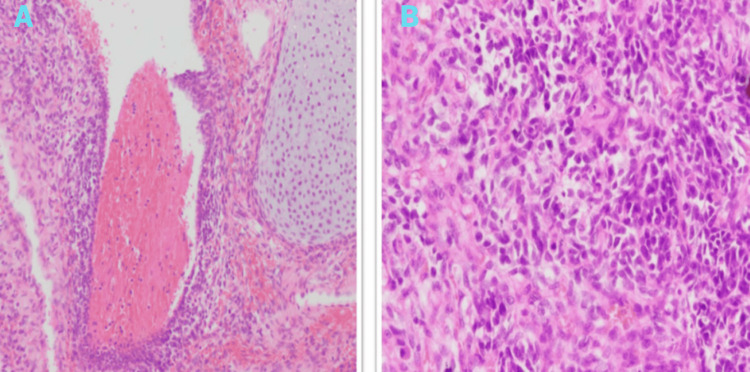
(A) Histopathology of PPB showing a mixture of primitive blastomatous and sarcomatous elements with cartilaginous differentiation. (B) Magnified view of PPB displaying primitive blastomatous elements, small hyperchromatic cells, and atypical mitoses. PBB, pleuropulmonary blastoma

The surgical specimen revealed a tumor lesion weighing 269 grams and measuring 6 × 4.5 × 6 cm. Histopathological examination demonstrated a combination of blastematous and sarcomatous characteristics, along with areas of bleeding, fibrosis, and necrosis. The mitotic proliferative index was high, as indicated by immunohistochemical staining (Figure 5). The final diagnosis was type II PPB.

## Discussion

PPB is an exceedingly rare thoracic tumor, representing less than 1% of childhood lung malignancies. These highly malignant tumors primarily affect children under the age of four [10]. Typically located at the periphery of the lungs, PPB may also involve the mediastinum, diaphragm, and pleura [11]. Its occurrence is slightly more common on the right side, with no significant gender preference. The symptoms are nonspecific and often mimic upper respiratory infections, including fever, cough, chest pain, and abdominal pain [12]. Many of these symptoms were observed in our patient.

Preoperative diagnosis of PPB is challenging due to its nonspecific imaging features. The tumor can appear as a pneumothorax or a large radiolucent lesion resembling a pneumatocele, complicating the distinction from other benign cystic lung lesions. A two-view chest X-ray may reveal a large mass with pleural effusion or chest wall invasion [13].

PPB primarily affects the lung’s outer lobes [14], but metastases to the brain, bone, and liver are possible in both type II and type III cases. Consequently, diagnostic imaging such as chest CT, brain MRI, and occasionally bone scans are essential. The association of PPB with DICER1 gene mutations, although characterized by low penetrance, explains the negative mutation test results in most patients. While over 80% of PPB cases show DICER1 mutations [15], only 15% of carriers develop tumors. Therefore, mutation analysis is recommended in suspected cases of DICER1-related disorders, although this may not be feasible in low-income countries due to the high cost [16].

Given the rarity of primary lung tumors in children, other potential causes of intrathoracic masses include congenital lesions or secondary tumors originating in organs such as the adrenal glands, thyroid, gonads, liver, kidneys, soft tissue, or bones [17]. Chest wall invasion may suggest conditions like rhabdomyosarcoma, undifferentiated sarcoma, or Ewing sarcoma. The tumor’s aggressive nature in types II and III is closely associated with the presence of solid components. Up to 25% of PPB cases present as extrapulmonary masses connected to the parietal pleura, highlighting the importance of considering primitive neuroectodermal tumors originating from the chest wall [18].

Type I PPB lesions can be mistaken for bronchogenic cysts, pulmonary cysts, pneumatoceles, and pulmonary interstitial emphysema. Differential diagnoses for locally aggressive type II and III PPBs include rhabdomyosarcoma, neuroblastoma, and Ewing sarcoma [19,20]. Although there is no confirmed transformation between PPB and congenital pulmonary airway malformation, some clinicians have noted potential associations between the two conditions [21,22].

Our final pathological results indicated that the PPB tumor exhibited partial differentiation into rhabdomyosarcoma, which is noteworthy. Rhabdomyosarcoma is known for its high glucose metabolism (average SUVmax of 7.2), which correlates with a poor prognosis [23]. However, the role of glucose metabolism in type II PPB cells remains uncertain. More research is required to determine if the rhabdomyosarcomatous differentiation observed in this case of type II PPB suggests a more aggressive form and worse prognosis.

Managing type II PPB requires a multidisciplinary team approach, involving pediatric oncologists, pulmonologists, surgeons, pathologists, and radiologists. This case highlights the importance of such collaboration in achieving accurate diagnosis, staging, and treatment [24].

The gold standard treatment for PPB in pediatric patients is complete surgical resection with clear margins, typically performed via thoracotomy [25]. Depending on the tumor’s extent, procedures such as lobectomy or segmentectomy may be required. However, tumors located in less common areas like the pleura, mediastinum, or diaphragm may not necessitate a standard lobectomy. The tumor’s fragility and its infiltration into nearby structures can complicate complete resection. If viable tumor cells remain after surgery and chemotherapy, pneumonectomy or lung irradiation may be considered, balancing the long-term effects of both options [26].

For inoperable tumors larger than 10 cm, biopsy followed by four to eight cycles of polychemotherapy based on the vincristine, actinomycin D, and cyclophosphamide (VAC) regimen is recommended, followed by extensive surgery to maximize tumor removal, according to the European Cooperative Study Group for Pediatric Rare Tumors (EXPeRT) [27]. Type I and Ir PPB are treated with complete excision and negative margins due to their low risk of metastasis, with adjuvant chemotherapy rarely necessary. In contrast, type II and III PPBs require a combination of chemotherapy and surgery, with radiotherapy considered if the tumor persists [28].

Currently, no specific molecular marker exists for the diagnosis of PPB [29]. Prognostic determinants, such as age at diagnosis, tumor size and location, distant metastases, and molecular markers - including mutations in the DICER1 gene, an autosomal dominant tumor suppressor - have been associated with PPB. Patients with suspected DICER1-related tumors should undergo testing for DICER1 gene mutations. Consequently, all cases of PPB should be referred for genetic counseling, as these factors profoundly impact the overall prognosis for type II PPB [30].

Type II PPB has a lower outcome rate compared to type III, despite both presenting significant morbidity and mortality rates. Patients with types II and III, who have better chances for a cure, require surgical intervention and chemotherapy. Surgical resection may delay progression to more advanced stages [31]. The prognosis remains grim, with overall survival rates of 62% for type II and 42% for type III PPBs at two and five years, respectively, despite multimodal therapy [32].

Worse outcomes are associated with cases of pleural, mediastinal, and extrapulmonary PPB. Tumor recurrence or metastasis following treatment is common, with two-year survival rates varying based on initial lung involvement, ranging from 11.3% to 80.0% [33,34].

## Conclusions

PPB is a rare and often aggressive cancer associated with a high fatality rate. In cases where a cystic or solid mass is detected in a child’s hemithorax without a clear etiology, it is essential to investigate all possible underlying conditions, whether benign or malignant, regardless of their rarity. CT scans play a crucial role in providing detailed information about the mass, facilitating assessment of its characteristics for a more accurate diagnosis and management.

Diagnosing PPB effectively requires a combination of imaging, histological, and clinical data. Treatment decisions must carefully differentiate PPB from more common benign congenital abnormalities, cystic lesions, and other malignancies. The age at which a patient is diagnosed with PPB, along with other determining factors, directly influences the prognosis. This case report illustrates that in low-income countries, successful treatment outcomes for such rare and challenging tumors can be achieved through appropriate management strategies.
